# Association between Education Level and Prognosis after Esophageal Cancer Surgery: A Swedish Population-Based Cohort Study

**DOI:** 10.1371/journal.pone.0121928

**Published:** 2015-03-26

**Authors:** Nele Brusselaers, Fredrik Mattsson, Mats Lindblad, Jesper Lagergren

**Affiliations:** 1 Upper Gastrointestinal Surgery, Department of Molecular medicine and Surgery, Karolinska Institutet, Stockholm, Sweden; 2 Department of Surgical Gastroenterology, Karolinska University Hospital, Stockholm, Sweden; 3 Division of Cancer Studies, King’s College London, London, United Kingdom; UT MD Anderson Cancer Center, UNITED STATES

## Abstract

**Background:**

An association between education level and survival after esophageal cancer has recently been indicated, but remains uncertain. We conducted a large study with long follow-up to address this issue.

**Methods:**

This population-based cohort study included all patients operated for esophageal cancer in Sweden between 1987 and 2010 with follow-up until 2012. Level of education was categorized as compulsory (≤9 years), intermediate (10–12 years), or high (≥13 years). The main outcome measure was overall 5-year mortality after esophagectomy. Cox regression was used to estimate associations between education level and mortality, expressed as hazard ratios (HRs) with 95% confidence intervals (CIs), with adjustment for sex, age, co-morbidity, tumor stage, tumor histology, and assessing the impact of education level over time.

**Results:**

Compared to patients with high education, the adjusted HR for mortality was 1.29 (95% CI 1.07–1.57) in the intermediate educated group and 1.42 (95% CI 1.17–1.71) in the compulsory educated group. The largest differences were found in early tumor stages (T-stage 0–1), with HRs of 1.73 (95% CI 1.00–2.99) and 2.58 (95% CI 1.51–4.42) for intermediate and compulsory educated patients respectively; and for squamous cell carcinoma, with corresponding HRs of 1.38 (95% CI 1.07–1.79) and 1.52 (95% CI 1.19–1.95) respectively.

**Conclusions:**

This Swedish population-based study showed an association between higher education level and improved survival after esophageal cancer surgery, independent of established prognostic factors. The associations were stronger in patients of an early tumor stage and squamous cell carcinoma.

## Introduction

Esophageal cancer is one of the most deadly cancers, with mortality rates of approximately 75–85% within 5 years of diagnosis.[1] Curatively intended treatment typically includes esophagectomy and is advocated in the 20–40% of patients with localized disease and acceptable fitness. Yet, only 30% of the patients who receive surgery survive the first 5 years after the operation, according to population-based research.[2,3] The incidence of esophageal adenocarcinoma is increasing rapidly in many Western populations, urging efforts to improve treatment and survival.[4] A higher socioeconomic status has been associated with health benefits and improved survival for some tumors,[5,6,7,8,9] but studies addressing esophageal cancer have failed to establish any association between socioeconomic inequality and survival.[10,11,12] The poor prognosis in esophageal cancer may be part of the explanation, since many patients die shortly after diagnosis. Moreover, even if socioeconomic variables are associated with survival, differences may have been missed due to limitations of power. Some studies indicate better survival after esophagectomy with a higher socio-economic status, yet other studies do not. [2,13] Education level is often used as proxy for socioeconomic status, since it is stable over the lifetime. In our previous study examining the role of education level in 600 patients operated for esophageal or gastric cardia cancer in Sweden between 2001–2005, an association between lower education level and higher mortality was indicated, but the power did not allow robust conclusions.[14] The objective of the current study was therefore to examine education level in relation to survival after esophagectomy in a substantially larger cohort of patients with esophageal cancer.

## Patients and Methods

### Study design

The design of this nationwide Swedish cohort study has been described previously.[2,15] In brief, this was a retrospective population-based cohort study including almost all patients who underwent curatively intended surgery for esophageal cancer in Sweden from January 1, 1987, through December 31, 2010, with complete follow-up for survival until February 2012. The cohort members were identified through Swedish nationwide health care registers. Relevant data were collected from nationwide registers and from surgery and histopathology records of all included patients. The study was approved by the Regional Ethical Review Board in Stockholm, Sweden, and the requirement for obtaining informed consent was waived since all data were analyzed anonymously.

### Study exposure—education

Information on education was collected from the Swedish National Education Registry, which was established in 1985 and is updated yearly with information on the highest formal education level attained by each Swedish resident.[16] The highest attained education level (fully or partially completed) at the time of esophagectomy was classified into three well-defined categories based on the Swedish National School of Administration and Statistics Sweden: 1) “compulsory” education, which corresponds to 9 years of education or less, including primary and lower secondary education (up to the age of 16 years), 2) “intermediate” education, corresponding to 10–12 years, including upper secondary education (the standard is 3 years), or 3) “high” education, represented by 13 years or more of formal education, including post-secondary education.

### Study outcome—mortality

Date and cause of death were collected from the Swedish Causes of Death Registry, which includes dates and underlying causes of all deaths among persons residing in the country, regardless of where they died (including abroad). Overall mortality up to 5 years after esophagectomy was the main outcome. Secondary outcomes were: 1) conditional mortality, defined as overall mortality up to 5 years after esophagectomy after exclusion of the first 90 days after surgery; 2) short-term mortality, including overall mortality within 90 days of esophagectomy; and 3) disease-specific mortality, defined as mortality associated with esophageal cancer according to the Causes of Death Registry (available only for deaths before January 1, 2011).

### Covariates

Covariates included the five most established clinical prognostic factors: age, sex, tumor stage[17], tumor histology, and history of comorbidity at the time of esophagectomy. Information on six comorbidities was collected: diabetes, cardiovascular disease, pulmonary disease, liver disease, renal failure, and other cancer. Comorbidities within the same group of these six comorbidities were counted only once. Information on covariates were collected through nationwide Swedish registers for in-hospital care and cancer, as well as from review of surgery and histopathology records from all Swedish hospitals ever having conducted esophageal cancer surgery during the study period.

### Statistical analyses

Cox proportional hazards models were used to assess the association between level of education and mortality, expressed as hazard ratios (HRs) with 95% confidence intervals (95% CIs). The proportional hazard assumption was evaluated by calculating the correlation between Schoenfeld residuals for the covariates and the ranking of the failure times. The correlations were close to zero and the p-values were above 0.05 which implies that the assumption was met. To manage partial missing data for level of education, tumor stage or tumor histology (21% of patients had at least 1 missing value) both complete case analysis and multiple imputations were conducted. The number of imputed data sets was 20 and monotone logistic method in PROC MI was used with the assumption that the missing data were missing at random (MAR).[18] The variables included in the imputation were age, sex, tumor stage, tumor histology, number of comorbidities, calendar period (time of surgery) and all-cause mortality. Furthermore PROC MIANALYZE was used to combine the results from the analyses of the 20 datasets. The underlying assumption was evaluated by means of sensitivity analyses using pattern-mixture models using the statement missing not at random.

The patient group with high education was used as the reference group. We performed 2 models, a crude (univariate) model only examining the association between education level and survival, and an adjusted (multivariate) model to correct for influence of other variables. The unadjusted survival rates for each educational level are visualized by means of a Kaplan Meier curve.

The multivariable regression model was adjusted for age (categorized into 3 equally-sized groups: ≤61, 62–70, or >70 years), sex (male or female), number of comorbidities (0, 1, or >1), tumor stage (0-I, II, III, or IV) [17] and tumor histology (squamous cell carcinoma or adenocarcinoma). Stratified analyses were performed for these 5 potential confounders and calendar period (time of surgery, divided into 3 groups: 1987–1994, 1995–2002, and 2003–2010). To evaluate if the estimates for education level changed over time, a regression model was used with the patient group with high education in the most recent calendar period (time of surgery) as the reference group. All stratified analyses were adjusted for the other variables included in the multivariable regression model. The statistical analyses were performed with SAS version 9.4 (SAS Institute, Inc., Cary, NC).

## Results

### Patients

In total, 1822 patients who underwent esophagectomy for esophageal cancer during the study period were included. Selected characteristics of the participants are presented in Table 1. Of all patients, 226 (12.4%), 629 (34.5%), and 898 (49.3%) had >12 years, 10–12 years, and ≤9 years of education, respectively. Education level was missing for 69 (3.8%) patients. The highly educated patients, as a group, were younger and presented more frequently with stage I tumors (27.9% versus 16.0% in the compulsory educated group). The education groups were more similar regarding distribution of sex, co-morbidity and tumor histology. During the first calendar period, a higher proportion of patients (39.4%) completed compulsory education only, compared to the most recent calendar period (25.8%) (Table 1).

**Table 1:** Patient and tumor characteristics and mortality after esophagectomy for cancer (n = 1822), categorized by education level.

	Level of education				
	***> 12 years***	***10–12 years***	≤ 9 years	Missing	***Total***
	*Number(%)*	*Number(%)*	*Number(%)*	*Number(%)*	*Number(%)*
Total	226 (12.4)	629 (34.5)	898 (49.3)	69 (3.8)	1822
***Age*, *in years***					
≤ 61	101 (44.7)	258 (41.0)	243 (27.1)	6 (8.7)	608 (33.4)
62–70	74 (32.7)	204 (32.4)	313 (34.9)	16 (23.2)	607 (33.3)
> 70	51 (22.6)	167 (26.6)	342 (38.1)	47 (68.1)	607 (33.3)
***Sex***					
Male	169 (74.8)	468 (74.4)	679 (75.6)	46 (66.7)	1362 (74.8)
Female	57 (25.2)	161 (25.6)	219 (24.4)	23 (33.3)	460 (25.2)
***Co-morbidity***					
0	109 (48.2)	312 (49.6)	467 (52.0)	51 (73.9)	939 (49.0)
1	80 (35.4)	217 (34.5)	293 (32.6)	15 (21.7)	605 (33.2)
>1	37 (16.4)	100 (15.9)	138 (15.4)	3 (4.4)	278 (15.3)
***Tumor stage***					
0–1	63 (27.9)	163 (25.9)	144 (16.0)	10 (14.5)	380 (20.9)
2	65 (28.8)	192 (30.5)	318 (35.4)	28 (40.6)	603 (33.1)
3	50 (22.1)	154 (24.5)	225 (25.1)	16 (23.2)	445 (23.6)
4	21 (9.3)	44 (7.0)	72 (8.0)	3 (4.4)	140 (7.4)
Missing	27 (12.0)	76 (12.1)	139 (15.5)	12 (17.4)	254 (13.5)
***Tumor histology***					
Squamous cell carcinoma	95 (42.0)	252 (40.1)	351 (39.1)	17 (24.6)	715 (39.2)
Adenocarcinoma	115 (50.9)	333 (52.9)	505 (56.2)	50 (72.5)	1003 (53.3)
Missing	16 (7.1)	44 (7.0)	42 (4.7)	2 (2.9)	104 (5.5)
***Calendar period (time of surgery)***					
1987–1994	49 (21.7)	158 (25.1)	354 (39.4)	52 (75.4)	613 (33.6)
1995–2002	74 (32.7)	239 (38.0)	312 (34.7)	8 (11.6)	633 (34.7)
2003–2010	103 (45.6)	232 (36.9)	232 (25.8)	9 (13.0)	576 (31.6)
***Length of follow-up*, *in days***					
*MedianInterquartile range*	787268–1825	553229–1449	436186–1105	440157–1280	757208–1315
***Mortality***					
90 days	12 (5.3)	64 (10.2)	121 (13.5)	11 (15.9)	208/1822 (11.4)
5 years	137 (60.6)	444 (70.6)	712 (79.3)	54 (78.3)	1347/1822 (73.9)
5 years, conditional*	125 (58.4)	380 (67.3)	591 (76.1)	43 (74.1)	1139/1614 (70.6)

* Conditional: excluding first 90 days after surgery

### Education level and mortality

#### All patients

**Fig 1:**
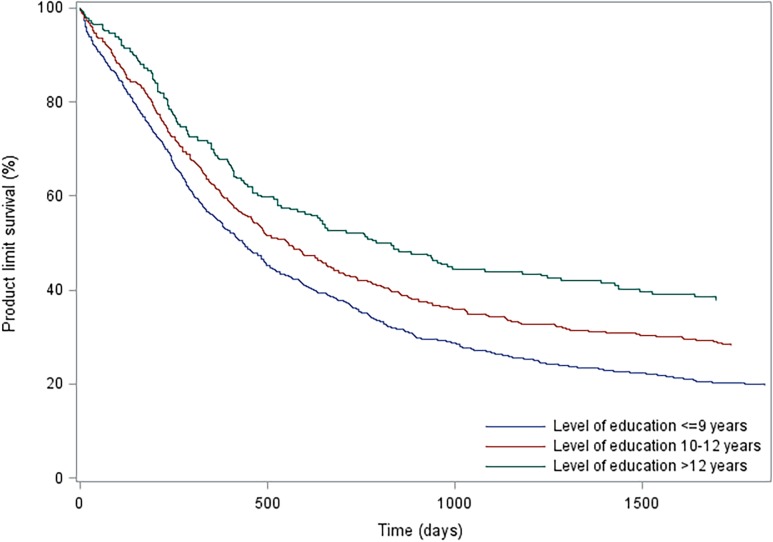
Kaplan Meier Curve of survival after esophagectomy for cancer, categorized by education level.

In total, 1347 (73.9%) patients died within 5 years of surgery, of whom 208 (11.4%) died within 90 days of surgery (Table 1). Of the patients who died within 5 years approximately 93.5% died from esophageal cancer (based on 975/1043 deaths that occurred before January 1^st^, 2010, when data on causes of death was available). Conditional 5-year mortality was 58.4% in the highly educated group and 76.1% in the compulsory educated group. The HRs for mortality after esophagectomy according to education level are presented in Table 2, and visualized in Fig. 1. Compared to the highly educated group, the overall 5-year mortality was increased in the intermediate educated group (adjusted HR 1.29, 95% CI 1.07–1.57), and in the compulsory educated group (adjusted HR 1.42, 95% CI 1.17–1.71). The conditional 5-year mortality HRs were similar, with corresponding adjusted HRs of 1.24 (95% CI 1.01–1.52) and 1.33 (95% CI 1.09–1.62), respectively. Also the 90-day mortality was higher in the intermediate educated group (adjusted HR 1.87, 95% CI 1.02–3.43) and the compulsory educated group (adjusted HR 2.28, 95% CI 1.26–4.11). The disease-specific mortality was similar to the overall mortality (data not shown).

**Table 2:** Cox regression models analyzing the association between education level and mortality after esophagectomy for cancer, using multiple imputation for missing values.

	Level of education		
	***> 12 years*** *(reference)*	***10–12 years*** *HR (95% CI)*	≤ 9 years*HR (95% CI)*
***Overall 5-year mortality***			
Crude model	1	1.31 (1.08–1.58)	1.63 (1.36–1.96)
Adjusted model*	1	1.29 (1.07–1.57)	1.42 (1.17–1.71)
***Conditional overall 5-year mortality*** ^*#*^			
Crude model	1	1.25 (1.02–1.53)	1.54 (1.27–1.87)
Adjusted model*	1	1.24 (1.01–1.52)	1.33 (1.09–1.62)
***Overall 90-day mortality***			
Crude model	1	1.90 (1.03–3.49)	2.54 (1.41–4.57)
Adjusted model*	1	1.87 (1.02–3.43)	2.28 (1.26–4.11)

Values are expressed as hazard ratios (HRs) and 95% confidence intervals (CIs).

* Adjusted for age (≤61, 62–70, or >70 years), sex (male or female), comorbidity (0, 1, or >1), tumor stage (0-I, II, III, or IV), and tumor histology (squamous cell carcinoma or adenocarcinoma).

^*#*^ Conditional mortality: excluding first 90 days after surgery.

#### Stratified analysis for age, sex and comorbidity

The adjusted stratified analyses for age, sex and co-morbidities are presented in Table 3. For age, the HR was increased for compulsory educated patients in older age groups, with HRs of 1.61 (95% CI 1.17–2.22) for patients of 62–70 years and 1.47 (95% CI 1.01–2.13) for patients older than 70 years.

**Table 3:** Cox regression models analyzing the association between education level and overall 5-year mortality after esophagectomy for cancer, stratified by patient and tumor characteristics, using multiple imputations for missing values.

	Level of education		
	***> 12 years (reference)***	***10–12 yearsHR (95% CI)***	≤ 9 years***HR (95% CI)***
***Age*, *in years***			
≤ 61	1	1.24 (0.91–1.67)	1.20 (0.89–1.62)
62–70	1	1.39 (0.99–1.94)	1.61 (1.17–2.22)
> 70	1	1.28 (0.87–1.90)	1.47 (1.01–2.13)
***Sex***			
Male	1	1.27 (1.01–1.59)	1.38 (1.11–1.71)
Female	1	1.37 (0.93–2.02)	1.53 (1.06–2.21)
***Co-morbidity***			
0	1	1.21 (0.88–1.65)	1.14 (0.84–1.54)
1	1	1.41 (0.87–2.30)	1.46 (0.90–2.35)
> 1	1	1.34 (1.01–1.79)	1.64 (1.24–2.15)
***Tumor stage***			
0–1	1	1.73 (1.00–2.99)	2.58 (1.51–4.42)
2	1	1.24 (0.89–1.74)	1.37 (0.99–1.89)
3	1	1.26 (0.90–1.76)	1.26 (0.91–1.74)
4	1	1.14 (0.68–1.93)	1.11 (0.67–1.83)
***Tumor histology***			
Squamous cell carcinoma	1	1.38 (1.07–1.79)	1.52 (1.19–1.95)
Adenocarcinoma	1	1.17 (0.87–1.59)	1.27 (0.95–1.71)

Values are expressed as hazard ratios (HRs) and 95% confidence intervals (CIs).*

* All values were adjusted for age (≤61, 62–70, or >70 years), sex (male or female), comorbidity (0, 1, or >1), tumor stage (0-I, II, III, or IV), and tumor histology (squamous cell carcinoma or adenocarcinoma).

For men, an increased HR was found for both intermediate educated patients (HR 1.27, 95% CI 1.01–1.59) and compulsory educated patients (HR 1.38, 95% CI 1.11–1.71). For the smaller group of women, the HRs were higher than in men, but the confidence intervals were wider, with HRs of 1.37 (95% CI 0.93–2.02) for the intermediate educated patients and 1.53 (95% CI 1.06–2.21) for the compulsory educated patients.

There was an increased 5-year overall mortality among less educated patients with comorbidity. Among patients with more than one comorbidity, the HRs were 1.34 (95% CI 1.01–1.79) for the intermediate educated group and 1.64 (95% CI 1.24–2.15) for the compulsory educated group (Table 3).

#### Stratified analysis for tumor stage and histology

As seen in Table 3, the adjusted HRs of mortality between education groups were largest in the tumor stages 0–1, with an HR of 1.73 (95% CI 1.00–2.99) in the intermediate educated group and 2.58 (95% CI 1.51–4.42) in the compulsory educated group, compared to the highly educated group.

There was a more pronounced increased HR of mortality for less educated patients with squamous cell carcinoma, with an HR of 1.38 (95% CI 1.07–1.79) for intermediate educated and 1.52 (95% CI 1.19–1.95) for compulsory educated patients, while the corresponding HRs among patients with adenocarcinoma were 1.17 (95% CI 0.87–1.59) and 1.27 (95% CI 0.95–1.71), respectively.

#### Trends over time

When highly educated patients operated during the most recent calendar period (2003–2010) were used as a reference, HR of mortality was increased in both previous calendar periods (Table 4). In all three calendar periods assessed, survival was higher in the highly educated patients compared to the lowest educated patients. The highest overall 5-year mortality was seen in the earliest calendar period, with HRs of and 2.52 (95% CI 1.81–3.53) and 2.78 (95% CI 2.04–3.78) for intermediate and compulsory educated patients, respectively.

**Table 4:** Cox regression models analyzing trends over time in the association between education level and overall 5-year mortality after esophagectomy for cancer, with the highly educated group operated between 2003 and 2010 as reference, using multiple imputation for missing values.

	Level of education		
	***> 12 yearsHR (95% CI)***	***10–12 yearsHR (95% CI)***	≤ 9 years***HR (95% CI)***
***Calendar period (time of surgery)***			
1987–1994	1.99 (1.29–3.05)	2.52 (1.81–3.53)	2.78 (2.04–3.78)
1995–2002	1.78 (1.18–2.66)	1.80 (1.30–2.47)	1.94 (1.42–2.65)
2003–2010	1 (reference)	1.50 (1.09–2.08)	1.37 (0.99–1.89)

Values are expressed as hazard ratios (HRs) and 95% confidence intervals (CI).*

* All values were adjusted for age (≤61, 62–70, or >70 years), sex (male or female), comorbidity (0, 1, or >1), tumor stage (0-I, II, III, or IV), and tumor histology (squamous cell carcinoma or adenocarcinoma).

Generally, the complete case analyses showed similar HRs to those based on imputed data for missing values, yet with less extreme point estimates and broader confidence intervals (results not shown). The disease-specific HRs were similar to the overall HRs (results not shown).

## Discussion

This study showed a clear association between lower education level and increased mortality after esophagectomy for cancer. These differences were especially apparent in patients with tumors of an early stage and of squamous cell carcinoma histology.

Strengths of this study are the population-based design, including virtually all patients operated for esophageal cancer in Sweden over more than 2 decades, long and complete follow-up of all study participants, and robust information on exposures, outcomes and established prognostic factors. Since esophageal cancer has a poor prognosis, and almost all patients die from cancer-related causes (>90%), we can be confident that the studied outcome mainly reflects disease specific mortality.

Education level is a frequently used and robust measure for socioeconomic status, which is relatively stable over time and easy to measure and compare between countries.[19] Other measures of socioeconomic equality, such as income and occupation-based measures are more complicated to measure, especially in older patients of whom a large proportion may be retired. Since healthcare is well organized, easily accessible and virtually without costs to everyone in Sweden, the access to healthcare is equal between patients, and should not influence survival.

A limitation of the study is that treatment and eligibility criteria for surgery change over time. However, we did stratify our results for calendar period effects (time of surgery), and found that higher education was associated with better survival than lower education in all three calendar periods, and overall survival appeared to be better in the most recent calendar period. It was also not possible to make smaller subcategories based on education level (e.g. bachelor or master level), because of the sample size and available data. Another limitation is that a relatively large proportion of patients had missing values for education level, tumor stage or histology. Therefore, we used multiple imputation methods to preserve statistical power. The HRs using the complete case strategy were similar to the HRs where imputation was used, which indicates no major influence of missing data on the results. For the complete-case analyses, we had to exclude a significant part of the cohort (i.e. all those with at least one missing value), which decreases precision and could have introduced selection bias if the missing mechanism was not “missing completely at random” (MCAR). To evaluate this further we also conducted multiple imputation with the underlying assumption of the missing mechanism is missing at random (MAR). The differences of the estimates between these two approaches could partly be explained that missing mechanism is not MCAR.

Although we adjusted our results for age, sex, tumor stage, tumor histology and number of comorbidities, other known and unknown patient, tumor and treatment related variables may also have confounded our results. The selection of comorbidities was decided upon before the data collection,[2] and did not entirely follow comorbidity severity scores such as the Charlson comorbidity index,[20] which uses slightly other definitions and categories. However, since comorbidities are not examined as main exposures, but as confounders, the potential impact of the used categorization on the results was considered limited. There are many potential explanations for an association between cancer survival and education level and other socio-economic factors, such as differences in comorbidity burden, lifestyle and health awareness, as well as choice of and adherence to treatment and healthcare seeking behavior.[5] Yet, as for chronic diseases such as cardiovascular disease and respiratory diseases, the association between educational inequalities and cancer mortality could be related, to a considerable extent, to certain health behaviors.[21]

Unfortunately, we did not have data on lifestyle factors, e.g. tobacco smoking, alcohol consumption, body mass index, and dietary factors. These factors might act as confounders or be part of the biological pathway from education level to association with survival. Especially smoking is known to be related to both socio-economic status and survival, yet body mass index, alcohol intake and physical activity can also mediate these effects.[21,22] Also in Sweden, lower-educated people are more likely to smoke than higher educated people, although this difference was more apparent in women compared to men.[23] A recent study suggested that although the contribution of smoking to socio-economic (educational) inequalities in survival is not negligible, this may be relatively limited in Sweden compared to other European countries.[24] In this cohort, the influence of severe comorbidity on survival, e.g. caused by tobacco or alcohol use, should also be limited because of the strict selection of patients eligible for esophageal cancer surgery. However, smoking and alcohol might have influenced our results, as shown by the stronger association between education level and survival in patients with esophageal squamous cell carcinoma, a cancer occurring more frequently in patients who are regularly exposed to smoking and alcohol.[25]

In contrast to previous studies addressing education in relation to prognosis after esophageal cancer surgery, including our previous study,[10,11,12,14] the present study showed a clear association, which was not explained by clinical prognostic factors. The larger sample size of this study might be one explanation, although it is not possible to establish a causal relation. Thus, despite the high mortality rates for esophageal cancer, factors associated with education level, for example lifestyle factors such as alcohol use and tobacco smoking or adherence to treatment and follow-ups, may substantially influence long-term survival after esophageal cancer surgery, although further sufficiently large studies are needed. Clinical implications might be that patients who are less educated may need guidance with lifestyle factors and a closer follow-up after discharge from hospital.

To conclude, this large and population-based study with adjustment for clinical prognostic factors showed a clear association between lower education level and increased mortality after esophagectomy, especially in patients with esophageal tumors of an early stage and squamous cell carcinoma histology.
